# Mr. Wainwright, on the Danger and Treatment of Pins Swallowed

**Published:** 1799-07

**Authors:** Thomas Wainwright

**Affiliations:** Dudley


### *To the Editors of the Medical and Physical Journal.*

GENTLEMEN,

IF you think the following cases worthy of a place in your miscellany, I shall be happy to see them in your next number, and am

Gentlemen,

Your obedient servant,

DUDLEY, *June* 9th, 1799. THOMAS WAINWRIGHT.

WILLIAM PHILLIPS, in the fortieth year of his age, was attacked with insanity, which continued two years. From the time of the recovery of his faculties, he complained of pain in the thyroid gland, with cough, a husky voice, and difficulty of breathing.—This gland was enlarged and sore when handled too freely. The difficulty of respiration very much resembled what takes place in the croup: the sound was like that of air drawn through a straitened passage. After some weeks the respiration became very laborious; the pulse at this time was small, weak, frequent, and irregular. Loss of appetite and of sleep; great prostration of strength, restlessness, delirium and convulsions closed the melancholy scene. The disease was evidently an inflammation of a part of the trachea. The repeated application of blisters and leeches to the seat of pain, and on which I had chiefly relied, availed nothing: in short no relief was obtained from the exhibition of medicine. The trachea being laid open after death; two pins without heads were found sticking, the one on the thyroid, and the other in the cricoid cartilage, opposed to each other, in an horizontal direction.* The trachea was much inflamed and ulceration had taken place round each pin. The ulceration had penetrated through the substance of the thyroid cartilage; the point of the pin pressing against a vesication which had arisen on the skin of the throat, a few hours before the patient's death, the second pin was working it's way by ulceration through the cricoid cartilage into the æsophagus. His friends could now recollect, that the deceased had, when insane, been in the habit of swallowing pieces of iron &c. and a mark was pointed out on the skin of the abdomen, where it was said an iron skewer, which he had swallowed, had ulcerated through. Had the pin, which stuck in the thyroid cartilage, been the only one, I believe that the patient might have survived: and that the pin would have escaped through the ulcerated opening, completed a few hours before his death, by the vesication.

If

* Mr. PHILLIPS, of Saville-House, Leicester-square, has a drawing of the trachea, with the pins in it.

If the cauſe of this extraordinary diſeaſe had been found out, and vomiting, coughing, and ſneezing artificially excited, had failed to diſlodge the pins, a longitudinal inciſion might have been made into the trachea, and the pins extracted by the forceps or the finger. A lady of my acquaintance has a cicatrix on the ſkin of the throat, occaſioned by the uſe of cauſtic, which ſucceeded in opening a paſſage for a pin, which had got into the trachea. How had the pins got into this patient's throat?—Had he, as they were without heads, thruſt them externally through the integuments?—or were they ſwallowed, and accidentally ſlipt into the trachea?—or had they ulcerated into the trachea from the æſophagus? The ſecond opinion is, perhaps, the moſt probable. It happened to a boy cracking nuts, that the ſhell of one ſlipped into the trachea: his life was ſaved by a violent fit of coughing, which expelled the nut-ſhell. A girl was brought into St. Thomas's hoſpital, in 1791 or 92, who was ſaid to have ſwallowed a cockle-ſhell. The whale-bone and ſponge was repeatedly applied to no purpoſe: an obſcure reſiſtance was thought to be felt in one part of the æſophagus. She died; and, upon diſſection, the cockle-ſhell was found in the trachea. The parts were preſerved, and are, I believe, in the collection of Mr. Ashley Cooper. A man, who had died ſuddenly in the ſtreet, was carried into St. Thomas's; his death was found to have been occaſioned by a piece of cheeſe having ſlipped into the trachea. There are grounds to ſuſpect two other caſes of diſeaſe in the trachea, which terminated fatally, to have ariſen from ſome extraneous body having fallen into it. Both were females: the one I attended nearly through the whole period of the diſeaſe. The ſymptoms were ſimilar to thoſe in the caſe of W. P. The remedies employed were of little, or rather of no ſervice. The other caſe occurred after the death of W. P. and the narration of the caſe led me to believe, that in this inſtance the diſeaſe originated from a pin having got into the trachea. This patient was in a dying ſtate when I ſaw her. Permiſſion to examine the trachea after death could not be obtained in either of theſe caſes. There was evidently, in both, inflammation and ulceration of a peculiar ſpecies in the trachea, differing totally from cancer; and from the very prevalent cuſtom, with the lower claſs of women, of putting pins into their mouths, there is ſome probability that one had ſlipt into the trachea.

In the Medical Muſeum, vol. 2. p. 496, three caſes are related, where extraneous ſubſtances had got into the trachea. The 1ſt, of a plumb-ſtone, diſcharged from the trachea by a violent cough, excited by the exhibition of a very acrid and ſtimulating remedy. In the 2d caſe, a pea was expelled by the emetic effect of a large doſe of oil. And a bone was diſcharged from the trachea in the third caſe by violent ſneezing, from ſnuffing powder of the lily of the valley into the noſtrils.

